# Synthetic and Bio-Artificial Tactile Sensing: A Review

**DOI:** 10.3390/s130201435

**Published:** 2013-01-24

**Authors:** Chiara Lucarotti, Calogero Maria Oddo, Nicola Vitiello, Maria Chiara Carrozza

**Affiliations:** 1 The BioRobotics Institute, Scuola Superiore Sant'Anna, Polo Sant'Anna Valdera, Viale Rinaldo Piaggio 34, Pontedera 56025, PI, Italy; E-Mails: chiara.lucarotti@gmail.com (C.L.); n.vitiello@sssup.it (N.V.); m.c.carrozza@sssup.it (M.C.C.); 2 Center for Micro-BioRobotics@SSSA, Istituto Italiano di Tecnologia, Pontedera 56025, Italy; E-Mail: chiara.lucarotti@iit.it

**Keywords:** human sense of touch, mechanoreceptors, artificial touch, bioartificial skin, bio-hybrid tactile sensors, MEMS, BioMEMS, fully-biological tactile sensors, tissue engineering, hydrogels, cell-culture

## Abstract

This paper reviews the state of the art of artificial tactile sensing, with a particular focus on bio-hybrid and fully-biological approaches. To this aim, the study of physiology of the human sense of touch and of the coding mechanisms of tactile information is a significant starting point, which is briefly explored in this review. Then, the progress towards the development of an artificial sense of touch are investigated. Artificial tactile sensing is analysed with respect to the possible approaches to fabricate the outer interface layer: synthetic skin *versus* bio-artificial skin. With particular respect to the synthetic skin approach, a brief overview is provided on various technologies and transduction principles that can be integrated beneath the skin layer. Then, the main focus moves to approaches characterized by the use of bio-artificial skin as an outer layer of the artificial sensory system. Within this design solution for the skin, bio-hybrid and fully-biological tactile sensing systems are thoroughly presented: while significant results have been reported for the development of tissue engineered skins, the development of mechanotransduction units and their integration is a recent trend that is still lagging behind, therefore requiring research efforts and investments. In the last part of the paper, application domains and perspectives of the reviewed tactile sensing technologies are discussed.

## 1. Introduction

The sense of touch allows humans to assess object properties, such as size, temperature, vibration and texture, and to detect slippage and measure grasping force during manipulation tasks. As in humans, the sense of touch in robots would contribute to representing the interaction between the artefact and its surrounding environment. As a consequence, advances in artificial skin-like sensory systems, capable of mimicking the human sense of touch, would enable several applications in the neuroprosthetics, humanoid robotics and wearable robotics domains.

Several types of artificial sensitive skin (both synthetic skin and bio-artificial skins) have been studied for a long time and a large number of transduction mechanisms have been reported in the literature. Transduction mechanisms consist in the conversion of one form of energy into another one. In the human skin, tactile transduction is a complex mechanism involving populations of mechanosensitive afferent fibres innervating the distal fingerpad and the skin with its different layers, including fingerprints [1–4]. The mechanoelectrotransduction (or mechanoneurotransduction) occurs when an external stimulus transfers energy to the human fingerpad, in contact mode (e.g., mechanical probing) or contactless (e.g., heat transfer via radiation), so to elicit sequences of electrical discharges that reach the brain via the afferent pathways [5] and code the stimulus in a perceptual form.

This papers reviews the recent advancements of microfabrication technologies towards the development of an artificial skin with embedded tactile sensors, via Micro Electro Mechanical Systems (MEMS) or hybrid artificial-biological microstructures (BioMEMS) that permit the design and fabrication of new miniaturized sensors made out of different materials and with integrated sensing and processing capabilities. BioMEMS and related devices for artificial skin can be fabricated with different classes of artificial, bio-artificial, and bio-engineered (both macromolecules and tissues) materials, such as microelectronics related materials (silicon and glass), plastic and polymeric materials (e.g., polydimethylsiloxane, PDMS), biological components such as proteins, cells and tissues [6]. In this landscape, artificial soft materials, as well as inherently soft bio-derived structures, play a crucial role for the development of new tactile sensing systems for potential future deployment in domains such as hand prosthetics: indeed, soft materials can increase the size of the contact area, thanks to their higher conformability, increase the contact friction coefficient (and thus the grasp stability), protect distributed embedded sensors which also provide better contact information, improve cosmetics, the latter being a relevant feature to enhance the final acceptability by the end user [7].

Biomimetics in the design of artificial fingers can go beyond the use of soft materials. Indeed, the effectiveness of employing soft materials is enhanced if anatomy and physiology of human fingers are considered. The soft and pulpy tissue that is present between the skeletal bone and the skin addresses several functions, such as dissipating mechanical energy during impacts and protecting the bone tissues from lesions; because of its softness and of the elastic nature of the skin, the pulpy tissue can conform to most uneven surfaces of commonly used objects; further, due to its viscoelastic nature, it dissipates strain energy that is induced during manipulation of rigid objects, thus stabilizing the interaction [8,9]. From this analysis it follows that making soft robotic fingers is of paramount importance for a safer, more stable and reliant interaction between the robot finger and the handled objects [10].

The objective of this paper is to review the state of the art of the design principles and technologies at the basis of bio-artificial sensing skins. Both “*bio-hybrid*” and “*fully-biological*” tactile sensing systems will be considered in the review. Prior to that, a brief overview on synthetic artificial sensitive skins and related transduction mechanisms is provided.

In this paper, we classify as bio-hybrid all the tactile sensors in which the transduction mechanism is based on synthetic sensors that are immersed in a bulk of biological/bio-engineered macromolecules and/or tissues: according to this definition, the biological component has the function of packaging and does not play any active role in the transduction mechanism, being involved in the transmission only of the mechanical stimuli from the environment to the sensitive area. Further challenging, we classify as fully-biological all the tactile sensors which use biologically-grounded mechanisms to achieve the transduction: in this case, synthetic components are not required for the core mechanotransduction processes.

This review paper is organized as follows: main anatomical and physiological characteristics of the human somatosensory system are briefly recapped in Section 2 for the sake of clarity. Next, state of the art of artificial sensitive skins is discussed in Section 3: Section 3.1 briefly illustrates technologies and transduction principles for the development of a synthetic artificial skin; bio-hybrid and fully-biological tactile sensors are thoroughly analysed in Sectitions 3.2 and 3.3, respectively. Section 4 discusses the reviewed state of the art, with attention to future application domains of bio-hybrid and fully-biological tactile sensors, and draws the conclusions with a wrap-up on the main concepts presented in the paper.

## 2. Human Sense of Touch

### 2.1. Definitions

The inputs to touch modalities include mechanical interaction, heat transfer, and various stimuli producing pain [11]. This review focuses on sense of mechanical touch. The human sense of mechanical touch encompasses several sensory capabilities that may be classified on the basis of the neural input source (e.g., cutaneous, kinaesthetic, haptic) [12–15]. The cutaneous sense is mostly mediated by sensory inputs from receptors embedded in the skin. This is mainly associated to physical contact with stimuli, and such mechanical stimulation is coded by skin receptors that project towards associated areas in the central nervous system (CNS). The kinaesthetic sense is mostly mediated by sensory inputs from receptors located within muscles, tendons and joints of the body. This is mainly associated to information about static and dynamic body postures. The haptic sense is mediated by combined sensory inputs from both the cutaneous and kinaesthetic sensory systems in order to perceive and guide actions within the real world.

### 2.2. Mechanoelectrotransduction of Tactile Information by Human Glabrous Skin and Mechano-Receptors

The human hand is covered with a relatively thick skin which provides protection from the external environment and mediates tactile sensation [16]. The human sense of touch responds to external stimuli by means of several receptors distributed in the skin, such as mechanoreceptors (for the response to mechanical stimuli), thermoreceptors (for the response to thermal stimuli) and nociceptors (responsible for the sensation of pain), that provide detailed and specialized tactile feedback. In the following, the focus will be on sense of mechanical touch (e.g., force, pressure, vibration).

The skin is composed of a series of folds, such as epidermis and dermis, and is capable of bending in prehension; folds are disposed so to allow stable grasp, together with subcutaneous fats that enable greater firmness in holding. Papillae are located at the interface of the epidermis and dermis underneath the dermal ridges [17,18], affecting the response of mechanoreceptors (located in the skin at different depths and densities) to mechanical stimuli. As a matter of fact, the geometry of papillae is important to increase the sensitivity of tactile sensation; when a force is applied, papillae amplify the stress and create zones of stress concentration [17].

Electromechanical properties of the human skin are related to the mechanotransduction mechanism, through which mechanosensory units transduce the mechanical stimulus into sequences of electrical discharges, which are then transmitted to the central nervous system for further processing [17].

**Figure 1.**
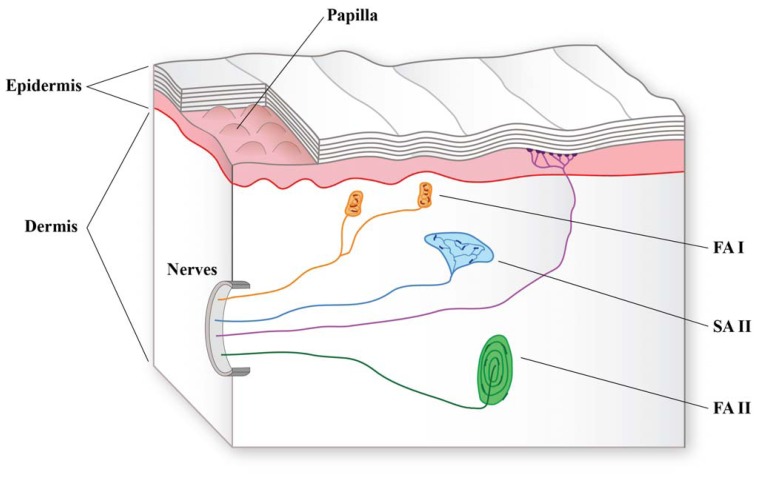
Section of the glabrous human skin illustrating the main classes of mechanoreceptors, comprising SA I (Merkel cells), FA I (Meissner corpuscles), SA II (Ruffini endings) and FA II (Pacinian corpuscles) tactile units (also, see Table 1).

The whorl region of the human fingerpad is characterized by a projected surface density of mechanoreceptors of about 240 units/cm^2^ [19], comprising Meissner's corpuscles, Merkel's discs, Ruffini endings and Pacinian corpuscles (Figure 1). These four classes of mechanoreceptors differ with regards to functional properties, such as sensitivity to static and dynamic events, size and structure of their receptive fields, number and densities within the separate subregions of the glabrous skin area and the particular perception that is elicited by firing events arising from each receptor type [20]. In electrophysiological experiments, the main classification of mechanoreceptors is based on the structure of receptive field and the rate of adaptation. Fast adapting receptors, named FA units, respond with burst of action potentials when the stimulus is applied and when it is removed; two types of fast adapting units are distinguished: FA I units (also named rapidly adapting RA units), which present a small receptive field characterized by several zones of maximal sensitivity distributed over a circular or oval area covering 5–9 epidermal ridges; and FA II units (also named Pacinian corpuscles, PC), which are characterized by a larger receptive field with only one zone of maximal sensitivity. Slowly adapting receptors, named SA units, remain active throughout the period during which the stimulus is in mechanical contact with its receptive field. Two different types of SA units are described: SA I units are characterized by several zones of maximal sensitivity (but not as many as FA I units) and an irregular discharge rate activated by a sustained stimulation, and SA II units, whose receptive field presents a single zone of maximal sensitivity and that show a regular discharge rate when they are probed with sustained stimulation [11,12,21].

SA I receptors are represented by Merkel's discs cutaneous mechanoreceptors, which are characterized by the simplest structure and enfold the non-myelinated ends of myelinated SA I axons [22]; they innervate the skin densely (about 70 units/cm^2^). The compressing strain from the skin in the distal half of the fingertip is transmitted by the Merkel's discs, which are critical for fine form detection and texture discrimination. Mansvelder and colleagues in [23] demonstrated that dissociated Merkel's discs are activated by hypotonic-evoked cell swelling triggering modifications of the concentration of Ca^2+^, that enables synaptic signalling to sensory afferents.

SA II receptors are Ruffini endings that are in the subcutaneous tissue (about 10 units/cm^2^). They are distributed more uniformly and can sense and perceive the stretch of the skin around joints and fingernails; they contribute to finger position and motion detection, perception of the shape of an object [16].

FA I receptors are represented by Meissner's corpuscles, innervating the skin even more densely (about 140 units/cm^2^) than SA I units; they are responsible of low-frequency vibration, flutter, slip and motion detection [22,24].

FA II receptors are Pacinian corpuscles (about 20 units/cm^2^) that are in the form of encapsulated endings: they present a specific, non-neural accessory structure surrounding the neural component and are responsible of high-frequency vibration detection [25]. The electrical response of Pacinian corpuscles, which is the proper transducer, to mechanical stimulation arises at the non-myelinated ending of the myelinated afferent nerve, and the mechanical energy that drives the transducer is transmitted through a fluid-filled lamellated capsule which serves as coupling between the external mechanical stimulus and the transducer. Some models of electrical response of Pacinian corpuscles to mechanical stimulations have been proposed by various research groups [26–28], with particular regards to the transmission of mechanical stimuli from the outer surface of the corpuscle to through the lamellar structure to the surface of the core.

### 2.3. Human Tactile Perception

Mechanoreceptors in the skin of the human hand are innervated by neural afferents (or first-order neurons) that transmit tactile information up to the primary somatosensory cortex. Spatiotemporal tactile information is encoded as spikes of action potentials, which are transmitted to the CNS via the spinothalamic pathway and the dorsal column-medial lemniscus pathway. The spinothalamic pathway transmits information to the thalamus about pain and temperature; the dorsal column-medial lemniscus pathway is responsible for transmitting pressure and vibration information from the mechanoreceptors to the primary somatosensory cortex. Johansson and Flanagan in [5] proposed that the somatosensory pathways could enable rapid classification of tactile stimuli by temporal-to-spatial conversion at level of second-order neurons (in the cuneate nucleus and spinal cord), which may function as coincidence detectors. A single primary afferent on the skin may project to about 1,700 cuneate neurons, each cuneate neuron receives signals from 300 cutaneous afferents, and about 11,000 cuneate neurons are engaged in classifying stimuli at each fingertip [5]. Then, information ascends up to the primary somatosensory cortex, which is divided into four main areas (1, 2, 3a and 3b). From these areas, tactile information (in the form of neural codes) is projected to the secondary somatosensory cortex and is decoded [29].

Cutaneous and kinaesthetic senses enable humans to discriminate between properties of objects. In particular, mechanoreceptors contribute to detection and perception of such properties of materials, such as form, force, texture, curvature, *etc.*

#### Force perception

Tactile feedback from the contact region contributes to perception of the employed force. SA I, SA II and FA I tactile units respond when a force is applied to the fingertip; the preferred directions of force are distributed in all angular directions with respect to the stimulation region. SA I units were observed to contribute to perception of tangential force components in the distal direction, SA II units in the proximal direction and FA I units in the proximal and radial directions [30].

#### Form perception

Form perception depends on surface structure and is defined by Johnson and Yoshioka in [31] as “perception of the specific geometric structure of a surface or object”. Such tactile attribute is affected by object's motion relative to the skin, by height of spatial features, by scanning velocities over 40 mm/s and by contact forces over the range 0.2–1 N.

#### Texture perception

Texture perception corresponds to “the subjective feel of a surface and depends on its distributed, statistical properties” (Johnson and Yoshioka in [31]). Texture perception consists of two main dimensions, such as roughness and softness. Considering grating stimuli, *roughness perception* is mainly determined by groove width, groove-to-ridge ratio and spatial period of the stimuli coming into contact with fingertips [32]. An individual variability among subjects in perceived roughness exists and such variability was associated to the use of different scanning velocities and forces [33]. Moreover, roughness was shown to be coded by spatial variations of discharges in SAI and SA II units, and its perception is enhanced when a tangential motion occurs between the skin and gratings of the tactile stimulus, whether active or passive touch protocols are used [34]. *Softness perception* is defined as “the progressive conformation to the contours of the fingers and hands in proportion to contact force” (Johnson and Yoshioka in [31]). The degree of softness is determined by the rate of growth of contact area with force and by the uniformity of pressure. Softness perception was shown to be correlated to the activity of SA I units because their rate of firing is highly correlated to the amount of deformation [31].

#### Curvature perception

Curvature is considered as “the rate of change in the angle of a tangent line to a curve as the tangent point moves along it” (Klatzky and Lederman in [11]). When small curved objects come in contact with the fingertip, SA tactile units provide a representation of the pressure on the skin and a single finger can discriminate between curved surfaces. Instead, if the curvature is larger, the surface is explored by multiple fingers. The firing rate of SA I and SA II tactile units is a function of vertical displacement and velocity and of the amount and the rate of change of curvature of the skin; however, SA I and SA II tactile units become silent with negative rate of change of curvature [12].

## 3. Artificial Tactile Sensing

**Figure 2.**
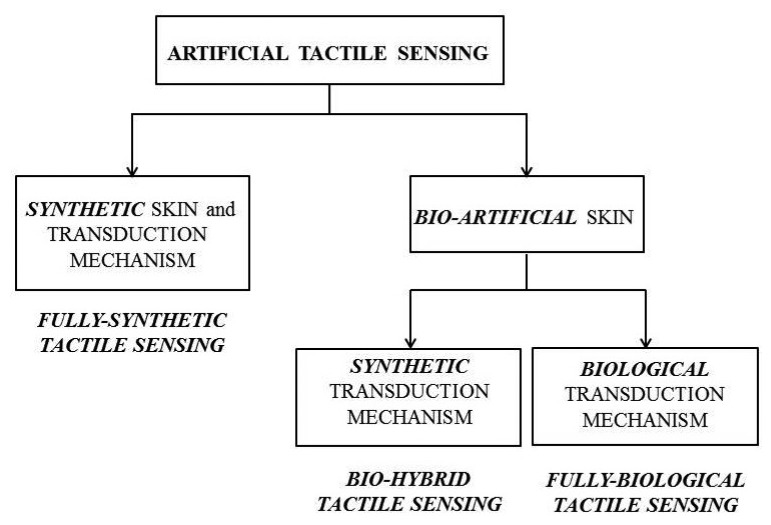
Classification of artificial tactile sensing with respect to the possible approaches to fabricate the outer interface skin layer and the transduction mechanism: synthetic *versus* bio-artificial.

In this section, the state of the art of artificial tactile sensing is analysed with respect to the possible approaches to fabricate the outer interface layer and the mechanotransduction units (Figure 2): synthetic skin and mechanotransduction versus bio-artificial ones. At first, with respect to synthetic skins, a brief overview is provided on various technologies and transduction principles that can be integrated beneath the skin layer. Second, the focus moves to approaches characterized by the use of bio-artificial skin as an outer layer of the artificial sensory system. Within this bio-artificial design solution for the skin, both synthetic and biological technologies and transduction mechanisms are presented.

### 3.1. Synthetic Skin and Transduction Mechanisms

Several reviews of tactile sensors have been proposed with respect to synthetic approaches for the design of the skin and transduction mechanisms [12,35–39]. Common synthetic tactile transduction techniques are based on capacitive, piezoelectric, piezoresistive, inductive, optoelectric and strain gauge methods (Table 2). The main characteristics associated with these techniques are briefly illustrated hereafter.

*Capacitive* sensors consist of two conductive plates with a dielectric material sandwiched between them. For parallel plates, the capacitance value is expressed as *C* = (*Aε_0_ε_1_*)/*d*, where *C* is the capacitance, *A* is the area of the two plates, *ε_0_* is the permittivity of free space, *ε_1_* is the relative permittivity of the dielectric material and *d* is the distance between the plates [9,40–45].

*Piezoelectric* sensors are composed of piezoelectric materials, such as poled ceramic lead zirconium titanate (PZT) and polyvinylidene fluoride (PVDF), which respond to the applied force/pressure [46–51]. Piezoelectric sensors are often named as *ultrasonic*, when using elements that emit an ultrasonic pulse within a medium (a rubber pad); the pulse is propagated through the medium and reflected. This echo pulse is then received by the emitting source. The transit time of the pulse is proportional to the thickness of the medium, therefore the strength of the echo pulse depends on acoustic properties of the medium and on the tactile stimulation conditions [52].

*Piezoresistive* sensors consist of a pressure sensitive element which changes its resistance upon applied force. The resistance value of a resistor having length *l* and cross-sectional area *A* is given by the equation *R* = *ρ*(*l*/*A*). According to this relationship, the resistance value is determined by both bulk resistivity *ρ* and dimensions, with dominant variational effects associated mainly to resistivity changes [53–56].

*Strain gauge* sensors consist of a resistive elastic unit, whose change in resistance is a function of the applied strain as shown by the equation *dR*/*R* = *Gε*, where *R* is the resistance, *ε* is the strain and *G* the gauge factor. If compared to piezoresistive sensors, the mechanotransduction properties of strain gauge devices are dominated by form factors rather than by variations in resistivity [57–60].

*Inductive* sensors are composed of a primary coil which induces a magnetic field sensed in a secondary sense coil; the mutual inductance between the coils modulates as a consequence of the applied load, thus modulating the amplitude and phase of the voltage measured in the sense coil [61,62].

*Optoelectric* sensors employ a light source, a modulatory medium, a transmission medium and a photodetector, the latter often in the form of camera or photodiode. Transduction occurs when changes in the tactile medium modulate transmission intensity, or the spectrum of the source light, as a consequence of variations in the applied force [63–66].

Packaging used in sensors for synthetic skin is mainly based on polymeric materials, such as silicone elastomers (e.g., polydimethylsiloxane in Dow Corning Sylgard 184^®^ PDMS, polyorganosiloxanes and silica in Smooth-on DragonSkin™ and Ecoflex^©^) and polyurethane rubbers (e.g., toluene diisocyanate and polyols in Polytek^®^ Poly-74 Series) [3,67]. Elastomers are useful due to their compliant nature, their resistance to temperature changes, mechanical toughness and “self-healing” properties. Also, the tensile strength and elongation at break of the elastomer permit it to withstand the stretch and abrasion due to forces encountered in object manipulation and exploration [68]. Moreover, the employment of polyurethane rubbers as synthetic substitutes for the skin is important in terms of thickness, elasticity and permeability.

### 3.2. Bio-Hybrid Tactile Sensing

Bio-hybrid tactile sensing systems consist of a synthetic sensor immersed into a tissue engineered skin (Figure 2, Table 3). Microfabrication technologies for synthetic sensors, such as photolithography, microfluidic patterning using microchannels, laminar flow patterning and microcontact printing, are used in order to generate patterns of cells on surfaces [69]. Also, MEMS technology allows the realization of array of synthetic sensors which can be incorporated into a tissue engineered skin, providing high spatial resolution and robust design: as a matter of fact, microfabricated devices, due to their chemical structure and surface properties, provide a platform where cells can adhere and be cultured, for biological and tissue engineering applications [70]. The robustness and advantages of this design approach are closely correlated to “self-healing” properties that may be shown by bio-hybrid tactile sensors, so to allow regeneration and repair of the skin or a damaged part by the cells themselves. As a consequence, a possible drawback is the need for microfluidic systems to manage nutrients and waste materials, and its complex integration with synthetic electronic circuitries.

Cheneler and colleagues in [71] and [72] proposed a design of a silicon-based bio-hybrid tactile sensor with integrated microfluidics and three local conductivity sensors. The tactile sensory system consists of microchannels etched into a silicon substrate providing cells with nutrients, oxygen and heat. Channels converge into a chamber with an array of micropillars allowing diffusion of nutrients whilst supporting a polycarbonate nanoporous membrane (100 μm thick); this membrane forms a layer upon which cells or tissues can be cultured and nutrients can diffuse through cells, therefore it is capable of sustaining viability of cells for a long time. Cells consist of tissue engineered alginate encapsulated fibroblasts, with mechanical properties comparable to the dermal layer of the human skin. Cells are confined to a well, *i.e.*, a cavity endowed with mechanisms to exchange nutrients and waste materials with the outer environment, using a PDMS cover plate acting as the epidermal layer. The transduction mechanism is provided by three local conductivity sensors, consisting of a pair of coplanar thin film metallic electrodes in close proximity, which are deposited on the membrane. The device is fabricated in three separate layers and then assembled. During experimental sessions, a constant current through the conductivity sensor is maintained and the voltage is measured in order to detect intracellular ion concentration changes. The system is designed to monitor response of different mammalian cell types when normal and tangential loads are applied by means of a mechatronic tactile stimulation platform for human active and passive touch studies [73].

Muhammad *et al.* in [74] proposed a conceptual design integrating silicon-based MEMS sensors with tissue engineered skin in order to measure the distribution of contact forces when the device comes into contact with stimuli. The tactile sensing system consists of a 4 × 1 linear sensor array and keratinocytes tissue engineered skin. Keratinocytes for tissue engineered skin are obtained from neonatal rat sacrificed by cervical dislocation and isolated by means of 0.25% trypsin and 0.02% EDTA and mechanical dissociation and cultured in 3:1 DMEH:Ham's F12 added with hydrocortisone, insulin, epidermal grow factor (EGF), fetal bovine serum (FBS), HEPES, L-glutamine as monolayers, with fibroblast feeder layer support. The appropriate number of keratinocytes is determined by means of a haemocytometer and then keratinocytes are seeded at a density of 0.2 × 10^6^ cells/cm^2^ onto Thincert TM membranes, grown in presence of mitomycin-treated feeder layer and cultured (at 37 °C with 5% CO_2_ and 100% RH) for one week on an alginate scaffold before being raised to the air-liquid interface for 2 weeks in order to allow keratinocytes stratification. The sensor is fabricated by means of MEMS microfabrication technologies and processes, such as photolithography, deep reactive ion etching, hydrofluoric acid release etching and gold thermal evaporation, so to engineer a sensor encompassing a clamped highly doped silicon membrane [75]. Then the sensor is mounted on a chip carrier and wire bonded in order to provide connections with electrical circuit: when a pressure is applied, the membrane deflects and a change in electrical capacitance between the two plates is measured by means of the detection circuit.

Buselli *et al.* in [76] proposed a polymeric flexible substrate with integrated bio-hybrid skin-like stretchable electrode for cell monitoring for the future development of a tactile sensor consisting of mammalian cells as core element. The electrode is composed of a bottom flexible substrate made of PDMS, on which a layer (90 nm) of Au is sputtered through a mask, so to obtain a gap (100 μm) between two conductors. An interlayer of Ti is used in order to improve the adhesion between Au and PDMS. A second PDMS layer is used to insulate the electrode; this layer is characterized by a central aperture in which cells are seeded and housed. In order to investigate cellular adhesion, 3t3 fibroblasts are seeded (4 × 10^5^ cells/mL) and incubated in Dulbecco's modified Eagle's medium added with fetal bovine serum (FBS) and penicillin-streptomycin (at 37 °C with 5% CO_2_ and 100% RH). Cells are stained with intracellular calcium indicator Oregon green Bapta-1 AM and visualized under a fluorescence microscope, before and after uniaxial stretching of the stretchable electrode to 1% strain for 1 minute. Results show that 3t3 fibroblasts attach sufficiently to the untreated PDMS substrate following one week of culture. When an external load is applied, an increase in fluorescence intensity and a following deformation are observed and this indicates an influx of Ca^2+^ within cells as result of the applied load.

Among bio-hybrid technologies, some research groups focused on BioMEMS devices fabricated by means of polymeric and elastomeric materials in order to provide substrates for cell adhesion and proliferation. Polymers, such as polydimethylsiloxane (PDMS), polymethylmethacrylate (PMMA), polycarbonate, polyurethane and polyester, are employed as materials for BioMEMS devices.

PDMS is one of the most used polymeric materials for BioMEMS devices thanks to its advantages in terms of rapid prototyping, transparency, elasticity, gas-permeability and adhesion to substrates. However, PDMS is not biodegradable; also, various strategies have been proposed and adopted to enhance its biocompatibility. To this aim, bioreactors consisting of microstructured PDMS have been fabricated for the perfusion of mammalian cells and for the cultivation of liver cells. Such bioreactors seem to be suitable for future applications in drug screening or tissue engineering. PMMA is another type of polymer employed in BioMEMS technology due to its transparency and biocompatibility (it is often used as substrate for mammalian cell culture) [70].

Among polyesters, glycolic and lactic acid-based poly(α-hydroxy acids) such as poly-L-lactide acid (PLLA) and poly-(lactic-co-glycolic acid) (PLGA) have been used for applications in drug delivery systems, scaffolds for tissue engineering, resorbable sutures, *etc.* Poly(glycerol-sebacate) (PGS) is a soft and elastic material that can be employed in tissue engineering applications (arteries, veins, nerves, *etc.*) thanks to its biocompatibility and bioresorbability. Poly(glycerol-sebacate)acrylate (PGSA) is composed of vinyl functional groups permitting the polymer to achieve a three dimensional crosslinked network, resulting in an increased polymer potential to encapsulate cells: as a matter of fact, PGSA is mainly used in the form of porous matrix for the encapsulation of stem cells. Also, polyurethanes are used for BioMEMS devices. They are employed as biomaterials for tissue engineering applications thanks to their good biocompatibility, biostability, and controllable and diverse mechanical properties. Among polycarbonates, the two main categories of biodegradable polycarbonates that have been investigated for biomedical applications are the co-polymers of poly(1,3-trimethylene carbonate) (PTCM) and tyrosine derived polycarbonates. PTCM is an amorphous elastomeric polymer; it seems to be rapidly degraded *in vivo*, therefore it is co-polymerized with other polymers [e.g., poly(D,L-lactide) (PDLLA) and poly(ε-caprolactone) (PCL)] in order to increase the degradability. Such co-polymers seem to be suitable for applications as scaffolds for tissue engineering [77].

Grayson and colleagues reported on technologies focusing on the improvement of the biocompatibility of implantable BioMEMS devices in order to obtain a better bioadhesion and cell proliferation. To this aim, the main techniques are microtexturing of surfaces, greater physical integration of the device and chemical modifications of surfaces. Absorption of peptides and proteins causes fouling of the device, therefore polymer-based modification methods (poly(ethylene) (PEG) or its analogues) are employed in order to inhibit protein adsorption and decrease biofouling. Also self-assembled monolayers methods are used to passivate the surface of the BioMEMS device and reduce biofouling [78].

### 3.3. Fully-Biological Tactile Sensing

In fully-biological tactile sensing, the outer interface layer consists of tissue-engineered, hydrogel-based and gelatin containing artificial skin (Table 4). Tissue-engineered skins are significantly used and refer to skin products consisting of cell cultures (extracellular matrix materials) or of combination of cells and matrices. Currently, one of the main application domains for bioartificial skins is dermal substitution or repair in case of ulcer or burned skin. Tissue-engineered substitutes offer tissue replacement without requiring a donor site and may produce better healing [79]. Scaffolds are designed to be implanted in patients as a template to restore or maintain original tissue functions [80,81].

Hydrogel-based artificial skin consists of engineered hydrogels, which are three-dimensional networks composed of cross-linked hydrophilic polymeric chains. Hydrogels are characterized by a high content of water and can undergo large change in volume by excluding or adsorbing water, so they can be cast into any shape, size or form. Hydrogels present several adequate features for biomedical applications, such as biodegradation, bioadhesion, bioactivity transport and mechanical properties [82,83]. Hydrogel encapsulation provides cells with a three dimensional environment similar to that experienced *in vivo* and may allow maintenance of normal cell function in order to produce tissues similar to those found in the body [84]. Alginate hydrogels can be used to encapsulate fibroblasts and keratinocytes can be cultured on the surface to form a bilayer structure [85]. Gelatin-containing artificial skin consists of gelatin that is obtained by a controlled hydrolysis of the fibrous insoluble protein, collagen, which is widely found in nature and is the major constituent of skin, bone and connective tissue. Gelatin consists of a unique sequence of amino acids (with a triple helical structure) and it is characterized by high content of them, including glycine, proline and hydroxyproline. Gelatin is known to exhibit the activation of macrophages and a high haemostatic effect; it is more convenient than commercially used collagen because a concentrated collagen solution is difficult to prepare from native collagen and also gelatin is more economical than collagen. Gelatin has been widely used as scaffold for tissue engineering, and in medicine as plasma expander, wound dressing, adhesive and absorbent pad [86].

Biological sensors may use live cells which maintain vital functions by responding quickly and with sensitivity to change in the external environment. Therefore, sensors using live cells as detector elements may be able to perform analyses faster and with more sensitivity than sensors using synthetic elements [87]. Moreover, cells respond to certain universal classes of effectors, such as hormones, that perturb basic metabolic functions; live cells are inherently sensitive to physiologically relevant concentrations of an effector [88].

Young *et al.* in [89] proposed the fabrication of polyHEMA artificial skin developed by means of polyHEMA-based hydrogel synthesized by UV-radiation inducing polymerization. HEMA and ethylene glycol dimethacrylate (EGDMA) are purified by distillation, and benzoin isobutyl ether (BIE) is used as UV-initiator; initial water addition (IWA) is added in order to determine surface and characteristics of the membrane. Then, mixtures of HEMA, EGDMA and BIE with water are molded between two glassy plates, whose inner surface is modified by adherence of a transparent polyethylene film. The mixture is then radiated for 20 minutes in an UV-reactor in order to polymerize. After polymerization, the product is soaked in water to remove chemical residuals and then polyHEMA membranes are kept in water to a saturated state and subjected to tests. The membrane is subjected to tensile strength measurements; experimental results show that the strength decreases by increasing amount of initial water added to the mix0ture; moreover, polyHEMA hydrogel is characterized by excellent water absorbing ability, wettability, complete transparency and dimensional change during water absorption and evaporation.

Choi *et al.* in [86] studied a gelatin-containing artificial skin by developing a gelatin-alginate sponge. The system consists of an absorbable gelatin-alginate sponge, which is prepared by a cross-linking method using 1-ethyl-3-(3-dimethylaminopropyl) carbodiimide (EDC) as cross-linking agent. Powders of gelatin and sodium alginate are dissolved in double distilled water (at 50 °C for 3 hours) in order to prepare 1 wt% solutions; each solution is mixed with 9:1, 7:3 and 5:5 weight ratios of gelatin to sodium alginate, and is stirred at room temperature for 30 minutes. The cross-linking degrees range between 10% and 35%. The sponge is immersed in 20 mL of acetone and slowly agitated at room temperature for 24 hours. Then, the water uptake ability is measured. The measurement of cross-linking degree is carried out by trinitrobenzenesulfonic acid (TNBS) assay and *in vivo* animal tests are performed in order to confirm the application of such gelatin-alginate sponge as a wound dressing material. Results illustrate that as the alginate content increases, porosity increases to 60% as well and water uptake ability is enhanced. Also, the drug release behaviour of the gelatin-alginate sponge loaded with two antibiotics (AgSD and GS) is analysed: results illustrate that such sponge shows sustained release behaviour for up to four days of elution period; the GS amount decreases by increasing the alginate content. The amount of drug release depends on the interaction between the antibiotic and the matrix of the sponge.

In another study, Choi *et al.* developed a gelatin-containing artificial skin by preparing a gelatin-hyaluronate sponge which is capable of mimicking the basic constituent of the human skin [90]. As in the previous case, an aqueous solution of gelatin and hyaluronic acid are dissolved in double distilled water (at 50 °C for 3 hours). Then, each solution is mixed with 9:1, 7:3 and 5:5 weight ratios of gelatin to hyaluronic acid, and is stirred at room temperature for 30 minutes. Ten grams of each solution are poured into polystyrene Petri dish and frozen and lyophilized; the obtained sponge is immersed in 20 mL of acetone-water mixture and, after slow agitation, it is withdrawn and washed with double distilled water, while magnetically stirred for 1 hour and then freeze-dried. Fourier-transformed infrared spectra, scanning electron microscopy (SEM) and stress-strain characterization (via the INSTRON machine) of the cross-linked sponge are used for the characterization procedure. Results show that the sponge presents a cross-linking degree of 10–35% gf/cm^2^, a mean pore size of 40–160 μm, porosity of 35–67% and a tensile strength of 10–30 gf/cm^2^. Moreover, porosity increases by increasing hyaluronic acid content, jointly with water uptake ability.

In a subsequent work, Choi *et al.* proposed a gelatin-containing artificial skin consisting of a cross-linked chitosan-hyaluronic acid sponge [91]. Mixtures of chitosan and hyaluronic acid are prepared by dissolving them in various ratios in 15% formic acid aqueous solution, in order to obtain 1 wt% solution. The obtained solution is filtered by means of a glass filter; by adding water to the solution, pH increases so much that precipitates are formed at pH 2–3. Then, precipitates are centrifuged and the obtained product is poured into a Petri dish and frozen; the Petri dish is then dipped into water in order to remove any chemical remains. The product is washed with water and frozen for one day and freeze-dried to prepare cross-linked sponges. The surface morphology is analysed by means of scanning electron microscopy and images show the sponge to be characterized by a uniform sponge structure with a narrow pore size distribution. The porosity ranges between 50–70% with average pore size of 90–160 μm. With respect to previous studies, chitosan-hyaluronic acid sponges present the poorest water uptake ability and they are less soft.

Hong *et al.* in [92] developed a gelatin-containing artificial skin consisting of hyaluronic acid sponge with and without antibiotic and epidermal growth factor (EGF). Powders of gelatin and hyaluronic acid are dissolved in double distilled water in order to obtain 1 wt% solutions. The solution is mixed with gelatin and hyaluronic acid in 9:1 weight ratio and then stirred at room temperature for 30 minutes. Ten grams of this solution are poured into Petri dish, frozen and lyophilized. Then the solution is immersed in acetone and slowly agitated. Finally, cross-linked sponges are washed with distilled water and sterilized. A cross-linked sponge is added with 200 mg of antibiotic solution and 3 mL of EGF solution are dropped on the sponge in order to damp the whole surface. Different types of sponges are applied on the dorsal skin defect of Wistar rat and on the postoperative days 5, 12 and 21 a skin wound tissue is biopsied. An immunohistochemical technique, using a monoclonal antibody to proliferating cell nuclear antigen (PC10), is applied. Sections of skin wound tissue are analysed by means of a microscope with charge-coupled device camera and measurements of re-epitheliazation during wound healing are carried out. The number of monoclonal antibody-positive cells is high in the case of the sponge with EGF at postoperative day 5, and then gradually decreases. The sponge containing antibiotics is characterized by the lowest number of proliferating cells at postoperative day 5; the highest proliferating ability is at day 12. The sponge containing antibiotic and EGF is characterized by a good proliferating ability at postoperative day 21 and presents the best wound healing properties (good performances on the whole for a healing period).

Lee *et al.* in [93] proposed a gelatin-containing artificial skin developed by means of porous gelatin scaffolds using a salt-leaching method. For the development of the scaffold, gelatin is dissolved in doubly deionized water and sodium chloride crystals are added at various weight ratios and then they are mixed. The solution is poured into a Teflon mold and dried in order to remove water. The morphology of the scaffolds is analysed by means of SEM microscopy and the mechanical strength is evaluated by INSTRON machine. Human fibroblasts are cultured in Dulbecco's Modified Eagle's Medium (DMEM) with addition of 10% FBS; cells are washed with PBS and then harvested by 0.05% trypsin-EDTA. Fibroblasts, after 1 week of *in vitro* culturing, present a good affinity to the scaffold and are distributed on the surface of pores; after 2 weeks of culturing, fibroblasts are proliferated on the whole area of the scaffold. *In vivo* study of cultured artificial dermal substitutes illustrates that an artificial skin containing fibroblasts is almost re-epithelialized and completely regenerated after 2 weeks of culturing.

Griscom *et al.* developed a PDMS microsystem with compartmentalized culture of three different types of cells in order to obtain a biological artificial skin [94]. Keratinocytes, dorsal root ganglia neurons (DGR) and spinal cord cells are used. The system consists of a glass substrate topped by a PDMS microsystem (with wells and microchannels) which is molded on a silicon substrate; it is fabricated by photolithography technology. Keratinocytes are placed in a 6 mm square central well; surrounding this well, DGR neurons are positioned in eight 2 mm diameter wells and spinal cord cells in eight 2 mm diameter wells. Wells are connected by means of microchannels of different width (200, 120, 80 and 40 μm). A second microsystem, encompassing sixty 20 μm diameter channels and DGR neurons and spinal cord cells cultured in 3 mm square wells, is developed. Surface treatments are carried out in order to allow wetting of microchannels and cell growth. Cell suspensions of neurons are prepared by trypsin treatment and mechanical dissociation of DGR neurons and spinal cord cells; skin cells are obtained by enzymatic and mechanical dissociation. Cells are cultured and, after 2 weeks, fixed with paraformaldehyde and stained with specific antibodies. Microsystems are first monitored in order to observe cell viability and propagation of neurons. In the first system, channel size greater than 40 μm allows keratinocytes migration to the neuron chambers and few neurons extensions extend to keratinocyte chamber: since keratinocytes are not localized in the skin chamber, there is not a chemical gradient creating signalization. In order to prevent migration of keratinocytes, wells are pre-wetted. The best results, permitting strong chemical signalization between chambers, are obtained with smaller channel widths. The patch-clamp measurement, an electrophysiological technique permitting the study of ion currents in cells, in response to external stimulation of the skin with capsaicin (active component of red chili peppers) in the skin compartment is carried out, and sodium and potassium channels are monitored on neuron cells in response to a sequence of depolarizing pulses. Initial patch-clamp recordings indicate a correlation of capsaicin in the skin compartment with an increase of Na^+^ peak current and conductivity.

Zacchi *et al.* presented a skin substitute composed of epidermal and dermal elements [95]. Keratinocytes are obtained from epidermis and fibroblasts from dermis. Human fibroblasts are seeded on squares of nonwoven meshes, three-dimensional scaffold of the benzyl ester of hyaluronan named HYAFF, and human keratinocytes are cultured on laser microperforated membranes (Laserskin). At confluence, pieces of Laserskin are fixed on nonwoven meshes on which fibroblasts have been grown for 2 weeks. The keratinocytes-fibroblasts cultures are placed on sterile stainless steel grids and cultivated for 2 weeks at air-liquid interface. Co-cultured cells are then processed with classic and immunohistochemical staining in order to determine the differentiation products of epidermal layer, basal lamina and extracellular matrix (ECM). Experimental results show that human fibroblasts and keratinocytes can be cultured on hyaluronic acid-derived materials; fibroblasts seeded inside three-dimensional structure of nonwoven mesh are able to adhere, proliferate and secrete ECM components; some of the most significant components of the membrane are produced and distributed at the boundary between epithelial and dermal layers and this indicates the formation of a basal lamina.

Lee and colleagues in [96] proposed an artificial skin composed of porous gelatin/β-glucan sponges. Gelatin and β-glucan are dissolved in doubly distilled water at 50 °C; solutions are poured into a polystyrene Petri dish, frozen at −70 °C and lyophilized for 24 hours. Sponges are cross-linked in acetone and then rinsed with distilled water to remove any residual. Finally, sponges are frozen, lyophilized and sterilized with ethylene oxide gas. After homogeneous mixing, 300 μL of gelatin/β-glucan solutions are dispersed into a 6-well tissue culture dish. Dishes are uncoated and coated with the mix0ture; cultured fibroblast cells are suspended in 100 μL of Dulbecco's Modified Eagle's Medium (DMEM) with 10% fetal bovine serum (FBS) and then spread on each sponge, incubated and added with fresh medium. After culturing the dermal equivalent for one week, sponges are lifted onto porous polycarbonate membrane of the culture insert and human epidermal keratinocytes are distributed in the dermal equivalent; the so obtained artificial skin is cultured at air-liquid interface for one week. Measurements of contact angle, cell attachment and proliferation are performed. Concerning the contact angle, gelatin shows low hydrophilic behaviour; instead β-glucan presents high hydrophilic behaviour on Petri dish. By increasing β-glucan content, the contact angle decreases and both materials are characterized by a similar contact angle. Cell attachment and proliferation improve by increasing the gelatin ratio in the mixture. An *in-vivo* study of the artificial skin shows that after one week the skin enhances re-epithelialization of a full-thickness skin defect.

Mao *et al.* in [97] developed an absorbable scaffold composed of gelatin and chitosan for an artificial skin. Aqueous solutions of gelatin and chitosan are prepared: they are mixed in 7:3 weight ratios and then stirred. A glutaraldehyde solution is added to cross-link; then, mixed solutions are poured into Petri dishes, frozen at −40 °C and lyophilized. The resultant sponge is washed with distilled water and freeze-dried; then, the final scaffolds are sterilized. Fibroblasts are isolated from dermis by sequential trypsin and collagenase digestion; these cells are cultured in Dulbecco's Modified Eagle's Medium (DMEM) containing 10% fetal bovine serum (FBS), L-glutamine and penicillin-streptomycin, and then incubated. At confluence, fibroblasts are harvested and subcultivated in the same medium. Keratinocytes are isolated from human skin from plastic surgical procedures, then isolated into a single cell suspension by treatment with trypsin and EDTA (at 37 °C for 30 minutes), resuspended and expanded in number in tissue culture dishes in a serum-free keratinocytes culture-medium kit. Subconfluent primary cultures are washed twice and incubated with trypsin-EDTA to detach cells. The effect of trypsin is then inhibited by adding the complete medium and cells are centrifuged and resuspended for reseeding and growing in new tissue culture dishes. Fibroblasts suspended in the medium are then seeded on the gelatin-chitosan scaffold in 24-well tissue culture dishes and cultured for 3 weeks. At confluence, keratinocytes are isolated into a single cell with a trypsin suspension (0.25%) and then the cell suspension is diluted with fresh culture medium to the final concentration and seeded directly on the scaffolds containing fibroblasts. After 2 days of incubation, the cell scaffold is transferred into 6-well tissue culture dishes and cultured in the keratinocyte culture medium. Fetal bovine serum (FBS) is added to the fibroblasts-keratinocytes co-culture. Water uptake ability and cell proliferation are analysed. Results show that the water retention is higher than 100% and it becomes higher by increasing gelatin ratio. The cell growth rate is related to thickness of the scaffolds: it decreases by decreasing the thickness. The obtained artificial dermis is flexible and the aggregation of chitosan chains in the scaffold permits to obtain a structure stable in size and shape.

Yang *et al.* in [98] proposed a tissue engineered artificial skin consisting of a layer of keratinocytes and a dermal matrix with type I collagen containing fibroblasts. Type I collagen is extracted from rat tail tendons; acid-extractable collagen is dissolved in acetic acid by stirring and the obtained collagen solution is separated from insoluble residue by centrifugation. Type I collagen is then purified by purification in NaCl and redissolution in acetic acid. Human skin cells are isolated from a circumcised neonatal foreskin. Dermis and epidermis are incubated in culture medium without serum (at 4 °C for 16 hours); fibroblasts are isolated from dermis by type I collagenase and are cultured in Dulbecco's Modified Eagle's Medium (DMEM) containing 10% fetal bovine serum (FBS). Instead, keratinocytes are isolated from epidermis by 0.25% trypsin solution. Then, cells are cultured in keratinocytes serum free medium. Collagen threats are prepared from type I collagen: a collagen solution in acetic acid is loaded in a peristaltic pump; a silicon tube is connected to a needle, immersed in a coagulation solution containing polyethylene glycol in sodium phosphate dibasic and sodium phosphate monobasic at pH 7.55, which is moved for threats production. Collagen gels on contact with the neutral pH solution and threats obtained are transferred into a solution bath containing sodium phosphate dibasic, potassium phosphate monobasic and NaCl. 9 threats are joined to 9 threats in order to obtain one layer of the mesh; then, 5 layers are stacked to obtain the mesh. The final product is then sterilized and cross-linked. In order to obtain a dermal equivalent, fibroblasts are suspended, harvested and stirred with a collagen solution. Cell-collagen mixtures are then positioned in tissue culture dishes and polymerization follows. After polymerization, fibroblasts are dispersed through the collagen gels and the mixture becomes the attached gel. Fibroblasts contract the collagen gel into a fibrillar connective tissue-like dermal equivalent. An *in vitro* artificial skin is produced by casting cell-collagen mixtures as dermal equivalent onto 3 μm porous polycarbonate membrane of the culture insert. After one week, the culture medium is removed and epidermal keratinocytes are applied to the surface. Then, it is submerged under keratinocyte culture medium inside and outside the culture insert. The developed multilayered artificial skin is cultured at air-liquid interface by removing inside medium and underlying culture medium is switched to keratinocytes medium with 10% fetal bovine serum (FBS). Morphology and immunohistochemistry examination is performed: results show that optimum behaviour of dermal equivalent is obtained by 3 mg/mL collagen solution and attached gel culture, which has a jumping effect of growth factor on cell growth at lag phase. Keratinocytes of artificial skin produce and secrete some structural proteins, such as cytokeratin 19 and involucrin.

Sabolinski *et al.* in [99] experienced an artificial skin in venous ulcers. The skin consists of human fibroblasts, obtained by enzymatic dissociation of a portion of tissue and cultivated in Dulbecco's Modified Eagle's Medium (DMEM) containing 10% newborn calf serum, and keratinocytes, obtained from explant outgrowths or dissociated cells. An acid solution of purified bovine type I collagen is neutralized, mixed with a suspension of dermal fibroblasts and cast into a culture insert. Movements of fibroblasts throughout collagen permit to condense collagen fibres, reducing volume of collagen and concentrating it to form a dense collagen lattice adherent to the porous membrane. Then, a suspension of dermal keratinocytes is seeded on the lattice, cultured by immersion in culture medium until a 4 cell layer covers the lattice. Subsequently, the level of culture medium is lowered to expose the epithelium to the air: this permits epidermis differentiation and formation of stratum corneum, a protective outer layer of skin which is capable of enhancing strength, handling characteristics and durability of tissue. The developed artificial skin is used in the treatment of patients with venous ulcers and results indicate that the cultured skin physically and biologically interacts with the wound and provides adaptable response and regenerative tissue. Therefore, this artificial skin is suitable for treatment of chronic and acute wounds.

Concerning the transduction mechanism, the analysis of the state of the art showed that such types of fully-biological artificial skins have not been fully integrated with a truly bioartificial transducer for the implementation of a sensing system that could be potentially either connected with the human afferent neural pathways (in neuro-prosthetics applications) or integrated into a robotic artefacts (in humanoids or wearable assistive devices). The quest for bioartificial receptors is a recent trend and some advances have just been shown on possible approaches for fully-biological transduction mechanisms, as it is the case of approaches with Merkel's cells [100].

Kim *et al.* studied the nerve-dependency of Merkel's cells proliferation in cultured human fetal glabrous skin, in order to determine whether such cells proliferate *in vitro* in the absence of systemic factors, blood vessel and intact nerves [101]. Tissues are obtained from human embryos of 56–82 day gestational age. Fetal digits are cultured in Dulbecco's Modified Eagles Medium, containing HEPES buffer, L-glutamine, bovine serum albumin, penicillin G, streptomycin and fungizone. Then, samples are floated in 12 well culture cluster dishes (1.5–2 mL of medium in each well). Cultures are maintained at 37 °C and the medium is changed every 2–3 days. Tissues are harvested after culturing for 1, 4, 7, 14, 28 days. Immunolabeling (by avidin-biotin immunoperoxidase and diaminobenzidine coloration technique) is carried out in order to demonstrate the presence of anti-CK20 immunoreactive Merkel's cells. Merkel's cells are observed in the dorsal region of the digit; PGP 9.5-immunoreactive nerves are observed in the dermis and in the periosteal areas. After 28 days of culture, the number of Merkel's cells has been maintained or increased. PGP 9.5-immunoreactive nerves decrease after one day and disappear after four days of culture. Merkel's cells *in vitro* proliferation suggested that such development is nerve-independent in the human fetal glabrous skin [101].

Nagase *et al.* developed an organotypic culture system of Merkel's cells by means of Merkel's cell-containing epidermal sheets embedded in collagen gel [102]. Nerve cells and Merkel's cell-containing epidermal sheets are cocultured in order to investigate the effect of nerve cells on the maintenance of Merkel's cells. Tissues are obtained from footpad skin of 4-week-old male Wistar rats. Dissected skin is incubated (4 °C) in Dispase I solution that permits the separation of epidermis and dermis. Therefore, epidermal sheets are cultured and PC-12 nerve cells are employed in order to investigate the effect of nerve cells on the maintenance of Merkel's cells within epidermal sheets. In addition, 3T3 fibroblasts, DJM-1 squamous cell carcinoma cells and KHm-1 melanoma cells are used to test the specificity of any nerve cell-induced effects. Merkel's cell-containing epidermal sheets are cultured and the medium is changed every two days. Collagen gel is poured into a dish (30 mm diameter) in order to form an acellular collagen gel layer; then, the acellular layer is covered with 0.5 mL of collagen gel embedded with five epidermal sheets. Cellular behaviour is investigated by histochemistry, immunohistochemistry, electron microscopy and enzyme-linked immunosorbent assay. Results showed that both keratinocytes and cytokeratin-20-expressing Merkel's cells are maintained up to two week. After two weeks, keratinocytes within the epidermal sheets underwent degeneration and Merkel's cells disappeared along with keratinocytes degeneration after three weeks. Cocultures of Merkel's cells and PC-12 nerve cells increased the number of Merkel's cells within the epidermal sheets. Epidermal sheets with PC-12 cells retained their structure better than those without PC-12 cells [102].

Shimohira-Yamasaki *et al.* described a culture method allowing Merkel's cells to interact with nerve cells [103]. Merkel's cells are isolated from footpad skin of Wistar rats and incubated in Dispase I solution (4 °C) permitting the separation of epidermis and dermis layers. Then, epidermal sheets are agitated in a flask containing trypsin solution (3 minutes, 37 °C), filtered and centrifuged. Merkel's cells and keratinocytes are obtained and incubated in culture dishes in a CO_2_ incubator (37 °C). The nerve cells NG108-15 (maintained in Dulbecco's Modified Eagle Medium added with 10% FBS, HT, NGF and NT3) and the nerve cells PC-12 (maintained in Dulbecco's Modified Eagle Medium added with 20% FBS, NGF, NT3 and penicillin) are employed in order to investigate their interaction with Merkel's cells. The behaviour is analysed by immunohistochemistry and electron microscopy. When nerve cells are added to the culture system, both nerve cells and Merkel's cells outgrew and synapsis-like structures appeared at their contact points. Nerve fibres promoted Merkel's cell survival, compared with keratinocytes only. The number of Merkel's cells with coculture of nerve cells is greater than that of Merkel's cells without cocultured of nerve cells at two days in culture [103].

Finally, fully-biological tactile sensing systems are 3D rather than mainly planar structures as it is the case of skin layers. As a consequence, to meet the metabolic needs for nutrient and oxygen delivery and waste removal, transport by diffusion is not sufficient but requires a convective pathway, (vasculature): various approaches have been proposed to this aim, including molding of PDMS [104] and angiogenesis [105].

## 4. Discussion, Application Domains and Conclusions

Robots and advanced hand neuro-prostheses are required to perform human-like manipulation tasks; therefore they should be equipped with artificial tactile sensory systems capable of mimicking the human sense of touch. This means that advanced robotic hands should be endowed with both an artificial sensing system capable to acquire the physical variables (e.g., contact force, pulpy tissue deformation map) underpinning the tactile information through physical interaction, and to translate—through multi-layered architectures and processing algorithms—the measured physical variables into one or more specific touch perceptions, such as force, form, texture (including roughness, and softness) and curvature [39]. This paper focused on the review of the state-of-the-art technologies at the basis of an artificial sensing system, while architectures and algorithms regarding data processing and the elaboration of artificial touch perceptions were out of the main scope of this work.

In order to obtain an artificial tactile sensory system, an outer interface layer with distributed and embedded tactile sensors should be developed. To this aim, in this review paper, the achievement of an artificial sense of touch has been analysed with respect to possible approaches to fabricate the system (Table 5): synthetic approaches (e.g., piezoelectric, piezoresistive, strain gauge, capacitive, inductive and optoelectric sensors) *versus* bio-artificial ones (here classified as bio-hybrid and fully-biological).

Advances in the development of synthetic tactile sensing systems have been presented by several research groups (here recapped in Section 3.1), while bio-hybrid (Section 3.2) and biological (Section 3.3) tactile sensors are a more recent and less established trend if compared to synthetic approaches.

Synthetic approaches are now paving the way towards their application in several domains, such as neural prostheses, bioinspired and biomimetic robotics, biomechatronic devices (for surgery, endoscopy, rehabilitation and assistance) and humanoid robotics. In all these application domains, the artificial tactile sensing system should cope with three main challenges [106]:

- development of effective and usable robotic and mechatronic systems;
- increased understanding of mechanisms implemented by different biological systems;
- effective interactions with biological systems.

Several synthetic examples were shown in the literature to allow artificial tactile coding of surface attributes during contact and relative motion between the stimulus and the artificial fingertip, therefore successfully addressing the points (i) and (ii) above.

With respect to point (iii), robotic systems for neural prostheses, humanoid robotics and wearable robotics application domains should work together with the final user. To this aim, with particular reference to neuroprosthetics, interfaces between the human user and the sensorized robotic system should be designed and developed [107]. In spite of several recent progresses [108,109], human-robot interfaces are currently one of the most relevant bottlenecks in the way towards the development of dexterous and sensorized hand prostheses, able to carry out different grasping tasks and to record information to be delivered to the user [110,111].

Biological and soft materials for skin-like sensorized surfaces could be highly relevant in prosthetic hand design because of the fact that a soft skin can protect embedded sensors, increase the size of the contact area in order to provide better contact information due to embedded sensors, and increase the contact friction coefficient, and thus ensure a higher stability in the grasp. Biodegradable elastomeric polymers are used in combination with MEMS in tissue engineering applications. Among elastomers, polyesters, polyurethanes and polycarbonates are employed in combination with MEMS in tissue engineering applications, due to their good biocompatibility, degradability, compliance and softness when presented *in vivo* [77]. Along this direction, sensors using cells as active elements could assume a significant role for robotic applications because living cells maintain life functions by responding quickly and with great sensitivity to changes in the external environment [112]. Microfabricated systems provide an excellent platform for culture of cells and are useful for investigation of cellular responses to various external stimuli [70]. These systems permit a spatial and temporal control of cell growth and stimulus by combining surfaces capable of mimicking complex chemistries and geometries of the ECM by means of microchannels that can regulate transport and exchange of soluble factors and fluids [113]. MEMS are appealing for biological applications, particularly for the possibility to tune their physical and chemical characteristics on the micrometer and nanometer scale. BioMEMS, integrating biological and synthetic components, would permit to obtain long-term *in vivo* sensing and a greater degree of biocompatibility for the sensor. The growing interest in combining biological components and microfabricated devices may lead the development of fully integrated MEMS-based devices capable to replace biological systems in the human body [78]. However, in the past most of MEMS devices have been fabricated on rigid substrates, while flexible and compliant substrates will be required in order to mimic and investigate biological systems and for enhanced biointegration.

Bio-hybrid and fully-biological tactile sensing systems may enhance the level of bio-mimicry of robotic fingertips: as a matter of fact, thanks to the use of biological components and tissue engineered tissues, the finger of a robotic hand is similar to the natural finger and could be better accepted, if compared to a finger composed of synthetic elements only, by the human subject.

In addition, particularly applying to neuroprosthetics, in fully-biological approaches for tactile sensing systems, the neuromorphic coding [114] of tactile information may be obtained directly by a neuro-inspired spike-based architecture, which could be based on the use of living mechanoreceptors and would enable to directly connect the artificial tactile system to the CNS, by minimizing the physical number of connections to communicate tactile information.

Finally, bio-hybrid and fully-biological tactile systems could show self-healing properties, which would permit to overcome intrinsic limitations of synthetic approaches. However, despite the vast number of scientific works addressing the development of engineered skin tissues to be used for encapsulating artificial sensors, the investigation of sensing technologies involving living mechanoreceptors in the transduction chain is lagging behind and needs more effort and investments. In parallel, the achievement of thick tissues with differentiated biomechanical properties is still an open research topic, that requires notable advancements in the capability to introduce vascular structures in the engineered tissue [115]. We foresee that in the next decade, starting from the vast know-how matured in the development of tissue-engineered artificial skins, which are mostly composed of keratinocytes and fibroblasts, new sensing technologies will be conceived based on the use of living mechanoreceptors immeresed in engineered pulpy tissue and interfaced with biocompatible MEMS/BioMEMS recording/processing/stimulation units.

## Figures and Tables

**Table 1.** Types of tactile units in the glabrous skin of the human hand, their density, and functional and morphological properties (synthesis from [20] and [5]).

		**Response to Sustained Indentation**	
		**FA**	**SA**
**Receptive Field**	**I**	**Meissner's corpuscle** Small receptive field with distinct borders Sensitive to high-frequency dynamic skin deformation (5–50 Hz) Insensitive to static force 140 units/cm^2^ Flattened, horizontal lamellae surrounded by a connective tissue capsule	**Merkel's disc** Small receptive field with sharp borders Sensitive to low-frequency dynamic skin deformation (<5 Hz) Sensitive to static force 70 units/cm^2^ Nerve ending structure
	**II**	**Pacinian corpuscle** Large receptive field with diffuse borders Sensitive to high-frequency vibrations (40–400 Hz) Insensitive to static force 20 units/cm^2^ Oval shaped	**Ruffini ending** Large receptive field with obscure borders Low dynamic sensitivity Sensitive to static force 10 units/cm^2^ Dendritic ending with elongated capsule

**Table 2.** Fully-synthetic tactile sensing: transduction methods, advantages and disadvantages (synthesis from [12,38,39]).

**Fully-Synthetic Tactile Sensing**			

Transduction method	Modulated Parameter	Advantages	Disadvantages
*Capacitive*	Change in capacitance	High spatial resolution Good frequency response Long term drift stability High sensitivity Low temperature sensitivity Low power consumption	Severe hysteresis Stray capacitance Complex electronics Noise susceptible
*Piezoelectric*	Strain (stress) polarization	Flexibility Workability Chemical stability Good high-frequency response	High temperature sensitivity Poor spatial resolution Dynamic sensing only Simple electronics
*Piezoresistive*	Change in resistance	Flexibility High spatial resolution Good sensitivity Low noise Low cost Simple electronics	Large hysteresis Low frequency response Low repeatability
*Strain gauge*	Change in resistance	Sensing range High sensitivity Low cost	High hysteresis Non-linear response Susceptible to temperature changes Design complexity
*Inductive*	Change in inductance	High sensitivity High dynamic range No mechanical hysteresis Linear response Physical robustness	Usage limited to nonmagnetic mediums Low spatial resolution Low repeatability Complex electronics
*Optoelectric*	Light intensity/spectrum change	High density High spatial resolution Capability to sense both shear and normal contact forces Immunity from EMI	Large size Rigidness Loss of light by microbending causing distortion of signal

**Table 3.** Bio-hybrid tactile sensing: biological component, transduction mechanism and main features.

**Bio-Hybrid Tactile Sensing**			

Method	Biological component	Transduction mechanism	Main features
*Silicon-based bio-hybrid tactile sensor with integrated microfluidics and conductivity sensors*	Polycarbonate nanoporous membrane (100 μm thick) forming a layer upon which cells (tissue engineered alginate encapsulated fibroblasts) are cultured	Three local conductivity sensors, consisting of a pair of thin film metallic electrodes deposited on the membrane	The system is capable of monitoring the response of cells when normal and tangential loads are applied
*Silicon-based MEMS sensors with tissue engineered skin*	Keratinocytes tissue engineered skin; keratinocytes are obtained from neonatal rat sacrificed by cervical dislocation, isolated and cultured for 2 weeks to obtain keratinocytes stratification	4 × 1 linear sensor array fabricated by means of MEMS microfabrication technologies, mounted on a chip carrier, wire bonded and connected to the electronics	The system is capable of measuring the contact force distribution when the device comes into contact with stimuli by means of load-unload indentation cycles
*Polymeric substrate with bio-hybrid skin like electrode*	3t3 fibroblasts are seeded, incubated and attached to a PDMS substrate	Electrode composed of a PDMS bottom layer, an interlayer and a PDMS upper layer with a central hole in which cells are seeded and housed	3t3 fibroblasts attach sufficiently to the PDMS substrate after one week of culture. When a load is applied, a Ca^2+^ influx is observed
*Polymeric and elastomeric materials in MEMS devices*	Polymeric and elastomeric materials used as substrates for cell adhesion and proliferation (e.g., mammalian cells, liver cells, stem cells)	The transduction mechanisms are mainly based on synthetic principles	The systems show an improved biocompatibility and biodegradability

**Table 4.** Fully-biological tactile sensing: types of artificial skin and main characteristics.

**Fully-biological tactile sensing**	
Artificial skin or Transduction Mechanism	Main Classes and Characteristics
*Hydrogel-based artificial skin*	*PolyHEMA-based hydrogel*, synthesized by UV-radiation induced by polymerization:excellent water absorbing abilitywettabilitycomplete transparency
	*Gelatin-alginate absorbable sponge*, prepared by a cross-linking method:porosity of 60%water uptake ability increases with the alginate content
*Gelatin-containing artificial skin*	*Gelatin-hyaluronate sponge*: water uptake ability and porosity increase with the hyaluronic acid contentcross-linking degree of 10–35% gf/cm^2^; porosity of 35–67%;pore size of 40–160 μm; tensile strength of 10–30 gf/cm^2^
	*Cross-linked chitosan-hyaluronic acid sponge*:porosity of 50–70%; pore size of 90–160 μmpoor water uptake ability
	*Hyaluronic acid sponge*, with and without antibiotics and EGF:sponge with EGF: proliferating ability at day 5sponge with antibiotics: proliferating ability at day 12sponge with antibiotics and EGF: proliferating ability at day 21
	*Porous gelatin scaffold*, using salt-leaching method:mechanical strength and biodegradation rate of the scaffold increase with porosity and are modulated by addition of saltfibroblast proliferate over the entire area after 2 weeks of culture
	*Porous gelatin/β-glucan sponge*:cell attachment and proliferation increase with the gelatin ratiowater uptake ability increases with the β-glucan content
	*Absorbable scaffold composed of gelatin and chitosan*, and culture of fibroblasts and keratinocytes: water retention increases with the gelatin ratiocell growth rate decreases with the thickness
*Tissue-engineered skin*	*PDMS microsystem with culture of three types of cells* (keratinocytes, dorsal root ganglia neurons and spinal cord cells):the best behaviour is observed in microsystems with smaller channel widths
	*Skin substitute composed of fibroblasts and keratinocytes*:fibroblasts and keratinocytes can be cultured on hyaluronic acid-derived materialsfibroblasts seeded inside three-dimensional structure are able to adhere, proliferate and secrete ECM components
	*Tissue engineered skin composed of keratinocytes and dermal matrix with type I collagen containing fibroblasts*:optimum behaviour of dermal equivalent is obtained by 3 mg/mL collagen solutionkeratinocytes produce and secrete some structural proteins, such as cytokeratin 19 and involucrin
	*Tissue engineered skin composed of human fibroblasts*, used in the treatment of patients with venous ulcers:the cultured skin physically and biologically interacts with the wound and provides adaptable response and regenerative tissue
*Merkel's cells*	*Merkel's cells proliferation in cultured human fetal glabrous skin*:Merkel's cells in vitro proliferation suggested that such development is nerve-independent in the human fetal glabrous skin
	*Organotypic culture system of Merkel's cells by of Merkel's cell-containing epidermal sheets embedded in collagen gel:*cocultures of Merkel's cells and PC-12 nerve cells increased the number of Merkel's cells within the epidermal sheets
	*Culture method allowing Merkel's cells-nerve cells interaction*:when nerve cells are added to the culture system, both nerve cells and Merkel's cells outgrew and synapsis-like structures appeared at their contact points

**Table 5.** Comparison between approaches for artificial tactile sensing: fully-synthetic, bio-hybrid and fully-biological tactile sensing. Advantages and disadvantages of each approach.

**Artificial tactile sensing**			

Approaches	Types	Advantages	Disadvantages
*FULLY-SYNTHETIC* *tactile sensing*	Capacitive sensors Piezoelectric sensors Piezoresistive sensors Inductive sensors Optoelectric sensors Strain gauge sensors	Physical robustness Greater sensitivity	Non self-healing properties Biocompatibility
*BIO-HYBRID* *tactile sensing*	Silicon-based bio-hybrid sensor with microfluidics and conductivity sensors Silicon-based MEMS sensors with tissue engineered skin Polymeric substrate with bio-hybrid skin-like electrode	Bio-mimicry Bio-inspiration Self-healing Greater softness and compliance	Conservation of living cells
*FULLY-BIOLOGICAL* *tactile sensing*	Hydrogel-based artificial skin Gelatin-containing artificial skin Tissue engineered skin Merkel cells	Bio-mimicry Bio-inspiration Self-healing Wettability Regeneration of tissues Greater softness and compliance Biocompatibility Biodegradability	Conservation of living cells Rejection
